# A multicenter trial on the predictors of different subtypes of hemorrhagic infarction after thrombolysis

**DOI:** 10.1038/s41598-024-76189-0

**Published:** 2024-11-30

**Authors:** Mohamed G. Zeinhom, Sherihan Rezk Ahmed, Ahmed Mohamed Kohail, Islam Fathallah Mohamed Kamel, ALshimaa Mahfouz Abdelrahman, Omar M. AL-Nozha, Mohamed Almoataz, Tarek Youssif Omar Youssif, Ahmed Mohamed Ali Daabis, Hossam Mohamed Refat, Ahmed Ahmed Mohamed Kamal Ebied, Ahmed Elbassiouny, Ahmed Zaki Omar Akl, Ashfaq Shuaib, Mohamed Ismaiel, Asmaa Ibrahem Desouky Mostafa Ibrahem, Mohamed Fouad Elsayed Khalil

**Affiliations:** 1 Neurology Department, faculty of medicine, Kafr El-sheikh university, Elgeish street, Kafr el-sheikh, Egypt; 2 Neurology Department, faculty of medicine, Al-Azhar university, ELmokhaim St., Cairo, Egypt; 3 Neurology Department, faculty of medicine, Tanta university, ELbahr St., Tanta, Egypt; 4 Radiology Department, faculty of medicine, ELminia university, ELgomhoria St., Minya, Egypt; 5 Medicine Department, College of Medicine, Taibah University, 23 ELhars st., Madina, Saudi Arabia; 6 Neurology Department, Saudi German hospital, Sharjah, United Arab Emirates; 7 Neurology Department, Burjeel medical city Abu Dhabi, Abu Dhabi, United Arab Emirates; 8 Neurology Department, Burjeel Royal Hospital Al Ain, Al Ain, United Arab Emirates; 9 Neurology Department, Faculty of medicine Zagazig university, 2 elgeish Et, Zagazig, Egypt; 10 Neurology department, faculty of medicine, Ain shams university, ELabbasia St., Al khalifa elmamon St., Cairo, Egypt; 11 Division of neurology, department of medicine, University of Alberta, Clinical sciences building, Edmonton, AB T6G 2R3 Canada; 12 Neurology Department, El-Sahel Teaching hospital, 2 Yossef Karam, El Sahel, Cairo, 11697, Egypt; 13 Neurology Department Al-Raml Hospital, Alexandria, Egypt; 14 Neurology Department, Saudi German hospital Madinah, 23 ELhars St., Madina, Saudi Arabia

**Keywords:** Alteplase, Hemorrhagic infarction, Egypt, Saudi Arabia, Middle east, Stroke, Neurological disorders

## Abstract

Worldwide, stroke is a leading cause of long-term disability in adults. Alteplase is the only approved treatment for acute ischemic stroke (AIS) and results in an improvement in a third of treated patients. Most studies evaluated the post-alteplase haemorrhagic transformation of brain infarction as a homogeneous entity but we evaluated the predictors of each subtype of haemorrhagic transformation of brain infarction. Our trial included 616 AIS alteplase-treated patients. We evaluated the ability of different risk factors, clinical presentation, and imaging features to predict different haemorrhagic transformation (HT) subtypes. HT was seen in 152 patients (24.7%), higher NIHSS, cardioembolic stroke and atrial fibrillation were independent predictors of all ECASS-based subtypes of hemorrhagic infarction, in addition, anterior-circulation stroke was an independent predictor of hemorrhagic infarction type 1 (odds ratio [OR], 11.04; 95% CI, 9.81 to 12.70; P-value > 0.001) and type2 (OR, 11.89; 95% CI, 9.79 to 14.44; P-value > 0.001), while older age was also an independent predictor of parenchymal hematoma type1 (OR, 1.312; 95% CI, 1.245 to 1.912; P-value 0.02). In AIS patients treated with alteplase in Egypt and Saudi Arabia, higher NIHSS, cardioembolic stroke and atrial fibrillation were independent predictors of all ECASS-based subtypes of hemorrhagic infarction; in addition, anterior-circulation stroke was an independent predictor of hemorrhagic infarction type 1 and 2, while older age was also an independent predictor of parenchymal hematoma type1. Trial registration: (clinicaltrials.gov NCT06337175), retrospectively registered on 29/03/2024.

## Introduction

Stroke is a leading cause of long-term disability in adults. It ranks as the second leading cause of mortality on a global scale. Developing countries bear a disproportionate burden of stroke, representing 66% of the total stroke cases worldwide^1^.

Although intravenous thrombolysis with alteplase significantly improves long-term outcomes after ischemic stroke, it may produce hemorrhagic transformation of the infarction, which occurs in up to 5% of the patients, increasing post-stroke mortality and morbidity^2^.

The risk of HT increases with large core infarction, older age, hyperglycemia, the use of anticoagulation, and the presence of small vessel disease or cerebral microbleeds. The presence of HT can lead to a significant increase in post-stroke mortality^3^.

Although many studies evaluated the prevalence and risk factors for HT in patients treated with alteplase and showed that the prevalence of HT in patients treated with alteplase globally was 32% and in East Asia was 35%^4^, very little information on factors that contribute to HT in the Arab population, in this study, our objective was to assess the factors predicting ECASS-based different subtypes of post-alteplase haemorrhagic transformation in patients from the Middle East who have experienced ischemic stroke.

## Methods

### Trial design

We conducted our prospective open-label trial between June 2022 and October 2023 after receiving the appropriate ethical committee approvals.

All methods were performed in accordance with the relevant guidelines, regulations and declaration of Helsinki.

### Participants

Our study included 616 first-ever AIS patients treated with alteplase within four and half hours of which 248 patients were recruited from Kafr el-Sheikh, 254 patients from Nasr City insurance hospitals in Egypt, and 114 patients from Saudi German hospitals in Madinah, Saudi Arabia.

Before inclusion in our study, we got formal written informed consent from all eligible patients or their first order of kin.

The study consisted of five distinct groups. The first group consisted of 464 patients who did not experience haemorrhagic transformation, the second group comprised 73 patients who had HT type1, the third group comprised 54 patients who had HT type2, the fourth group comprised 14 patients who had PH type1, and the fifth group comprised 11 patients who had PH type2.

Our study adheres to CONSORT guidelines and includes a completed CONSORT checklist as an additional file.

### Inclusion criteria

We enrolled individuals of both genders, aged 18 to 75 years, who presented with their first-ever acute ischemic stroke and were eligible to receive alteplase as they presented within 3- to 4.5-hours, without a history of prior stroke in the past three months, NIHSS score ≤ 25, not taking any oral anticoagulants in the past 24 h, and without imaging evidence of intracranial hemorrhage^5^.

The diagnosis was made based on clinical history, physical examination, and specific findings from brain imaging. The study did not exclude patients who had experienced previous transient ischemic episodes (TIA). Only patients treated with alteplase were included in the study.

We excluded patients > 75 years old as advancing age is associated with an increased risk of HT, as well as worse stroke outcomes. Older patients experience an increase in systemic inflammation and blood-brain barrier (BBB) permeability and have a more significant burden of cerebrovascular disease, hypertension and diabetes, which induce inflammation and atherosclerosis, so if a stroke occurs, the age-related inflammation produces blood-brain barrier disruption leading to an increased risk of HT^6,7^.

Also patients older than 75 years old have higher possibility of atrial fibrillation which necessitates anticoagulation and limits the use of alteplase^8^.

### Exclusion criteria

Patients excluded from the study were those who had contraindications to alteplase such as patients whose CT of the brain revealed an acute intracranial hemorrhage, patients who had a prior ischemic stroke within 3 months, patients with recent severe head trauma within 3 months, patients with post-traumatic infarction that occurs during the acute in-hospital phase and AIS patients and a history of intracranial/spinal surgery within the prior 3 months, patients who had a history of intracranial hemorrhage, Patients who had a structural gastrointestinal malignancy or recent bleeding event within 21 days of their stroke event, patients who had platelets < 100 000/mm3, INR > 1.7, aPTT > 40 s, or PT > 15 s, patients who received a full treatment dose of LMWH within the previous 24 h, patients who received direct thrombin inhibitors or direct factor Xa inhibitors, patients who had symptoms consistent with infective endocarditis, and patients who had AIS who harbor an intra-axial intracranial neoplasm^5^. We ruled out patients who did not receive the complete dose of alteplase and those who had a documented medical history of chronic or recurring central nervous system disorders such as epilepsy, meningioma, multiple sclerosis, or a history of head trauma resulting in lasting neurological impairment.

We ruled out Patients who had major organ failure, ongoing malignancies, or a myocardial infarction during the preceding six weeks.

We excluded pregnant and lactating patients and individuals with stroke resulting from venous thrombosis or stroke following cardiac arrest or severe hypotension.

### Study procedures

The data collected encompassed many demographic and clinical variables, including age, sex, medical history of hypertension (HTN), ischemic heart disease (IHD), hyperlipidemia, diabetes mellitus, cigarette use, treatment history and the duration between symptom onset and treatment initiation.

The diagnosis of ischemic stroke was established through a detailed clinical history and examination and suitable brain imaging. All of our patients had CT of the brain and CT angiography (CTA) including the aortic arch through the circle of Willis before thrombolysis; the CT of the brain was performed on the 64-slice dual-source spiral CT scanner of Somatom definition by Siemens, and the supratentorial compartment scans were imaged with 5–8 mm contiguous sections and the brain stem and cerebellum scans were imaged with 3–5 mm slices, and we obtained eighteen images for each series. After thrombolysis, all of our patients underwent an MRI brain on a 1.5 T (Siemens Essenza) MR system, stroke protocol: T1W, T2W, fluid attenuation inversion recovery imaging (FLAIR), diffusion-weighted imaging (DWI), T2 Echo Gradient, and MRA brain & neck time of flight (TOF) if CTA was contraindicated and in our study 33 (5.4%) patients underwent MRA (TOF) brain& neck instead of CTA as they had renal impairment; we performed an additional brain CT scan after 24–36 h to evaluate hemorrhagic transformation^9^, and we considered hemorrhagic transformation symptomatic if the NIHSS score increased by 4 points or more^10^.

The computed tomography (CT)/ magnetic resonance imaging (MRI) examinations were assessed in our study by two highly experienced professionals: a senior stroke physician and a senior radiologist.

Large-vessel stroke was defined according to the Trial of Org 10,172 in Acute Stroke Treatment (TOAST) classification when patients had clinical and brain imaging findings of > 50% stenosis or occlusion of at least one of the following arterial segments on computed tomography angiography (CTA) or magnetic resonance angiography (MRA) if CTA was contraindicated: intracranial part of internal carotid arteries, middle cerebral arteries (M1/M2), intracranial portion of vertebral arteries, and basilar artery, and patients had cortical or cerebellar lesions and brain stem or subcortical hemispheric infarcts greater than 1.5 cm in diameter on CT or MRI, and there were no potential sources of cardiogenic embolism^11^.

We diagnosed the Small-vessel stroke according to TOAST classification when the brain imaging showed a brain stem or subcortical lesion which measured < 1.5 cm in its largest diameter, and the patient did not have a cardiogenic embolism or ipsilateral arterial stenosis of ≥ 50%^11^.

Posterior-circulation stroke (PCS) was defined as symptomatic ischemia occurring in the vascular area of the vertebral, basilar, or posterior cerebral arteries. Anterior-circulation stroke (ACS) manifests symptomatic ischemia within the internal carotid, middle, or anterior cerebral arteries; in addition, we assessed the baseline Alberta Stroke Program Early CT score (ASPECTS) of all patients included in our study, which is a 10-point quantitative topographic CT scan score used in patients with middle cerebral artery (MCA) stroke. A segmental assessment of the MCA vascular territory is made, and 1 point is deducted from the initial score of 10 for every region involved: caudate, putamen, internal capsule, and insular cortex; in posterior circulation stroke, we used pc-ASPECTS which is a 10 point scale, where points are lost for each region affected, thalami (1point each), occipital lobes (1 point each), midbrain (2 points), pons (2 points), and cerebellar hemispheres (1 point each)^12^. Questionable imaging is imaging whose ASPECT score disagreed with the senior neurologist’s and the senior radiologist’s, and the disagreement was settled by reaching a consensus decision.

In our study, we divided cerebrovascular vessels into segments: supra-clinoid internal carotid artery, first-division middle cerebral artery (M1), second-division middle cerebral artery (M2), first-division anterior cerebral artery (A1), second-division anterior cerebral artery (A2), basilar artery (B.A.), intracranial vertebral artery (V.A.), first division posterior cerebral artery P1), and second division posterior cerebral artery (P2). If one or more vascular segments were occluded and the patient was eligible for endovascular management, pre-stroke mRS score of 0–1^2^; causative occlusion of the internal carotid artery or MCA segment 1 (M1)^3^; age ≥ 18 years^4^; NIHSS score of ≥ 6^5^; ASPECTS of ≥ 6; and Ref.^6^ treatment can be initiated (groin puncture) within 6–16 h of symptom onset^2^. Then, the procedure was done by a senior neuro-intervention consultant using Philips Biplane Allura Xper FD20/15 release 8.2 with X-ray generator 100 kva, and the procedure was performed under general anesthesia in an angiography suite with biplane digital subtraction and road-mapping capabilities.

In the non-HT group, 27 patients were eligible. They underwent endovascular management, while in the HT groups, ten patients (six patients in the PH-1 and four patients in the PH-2) were candidates for endovascular management but did not undergo endovascular management due to the occurrence of hemorrhagic transformation and sudden deterioration of patient consciousness level just after receiving alteplase^13^.

All the patients underwent trans-esophageal echocardiography, 12-lead routine ECG 24, 24-hour continuous cardiac rhythm monitoring, carotid duplex, and blood pressure assessment. We diagnosed acute hypertensive response when the systolic blood pressure (BP) ≥ 140 mm Hg or diastolic (BP) ≥ 90 mm Hg detected on 2 recordings taken 5 min apart within 24 h of stroke symptom onset^14^, renal function, liver functions, coagulation profile, complete blood count, fasting, postprandial blood sugar, and HbA1C, and we diagnosed diabetes when fasting plasma glucose level was more than 126 mg/dl, or casual plasma glucose was more than 200 mg/dl, or HbA1C was more than 6.5^15^. Admission hyperglycemia was diagnosed when admission blood glucose value was more than 140 mg/dL^10^. We assessed the lipid profile on admission and diagnosed hyperlipidemia when blood cholesterol was more than 200 mg/dL; triglycerides were more than150mg/dL, LDL-cholesterol was more than100 mg/dL and/or HDL-cholesterol is less than 40 mg/dL)^16^. We measured the temperature of all of our participants orally at admission and twice daily during hospitalization (Temp Plus II, Ivac, Giessen, Germany) and diagnosed fever when at least one measurement was ≥ 38ºC (sub-febrile temperature 37.6–37.9 ºC)^17^.

Patients who experienced fever underwent a chest radiograph and urine analysis. If patients had specific symptoms, we performed cultures for sputum, stool and repeated blood cultures, especially if patients had fevers of unknown origin. We managed patients with fever using one gram of acetaminophen early after hospitalization and specific antibiotics according to culture and sensitivity tests^17^.

Based on AHA/ASA guidelines, inclusion and exclusion criteria for alteplase were established; 0.9 mg/kg of tPA up to a maximum dose of 90 mg was administered intravenously to eligible individuals within 4.5 h of the beginning of their clinical manifestations (10% bolus, 90% infusion in 1 h). After receiving IV-alteplase, all patients continued their management and rehabilitation in the stroke unit.

Hemorrhagic transformation was defined according to the European Cooperative Acute Stroke Study (ECASS) as any intracranial hemorrhage on any post-treatment imaging after thrombolysis start and increase of NIHSS by 4 points from baseline or death and classified into hemorrhagic infarction type 1 and 2, and parenchymal hemorrhage type 1 and 2^4^.

#### Primary endpoint

We evaluated the haemorrhagic infarction (HI) type1 predictors at 0 to 36 h after IV alteplase.

#### Secondary endpoint

We evaluated the Predictors of HI type2, parenchymal haemorrhage (PH) type1, and type2 at 0 to 36 h after IV alteplase.

### Sample size

We employed G-power software to calculate the power of our sample size, which was 90%, given a two-sided confidence level of 95% and an alpha error of 5%.

### Randomization

We included the cohort of patients who met our inclusion criteria and agreed to participate in the trial and presented to the emergency of the prespecified hospitals during the time period from June 2022 to October 2023, we did not use randomization plan as all the patients received the same treatment.

### Statistical analysis of the data

We used the IBM SPSS software package, version 20.0 (Armonk, NY: IBM Corp.), to analyze our data and base all efficacy analyses on the per-protocol analysis principle. Both the primary and secondary outcomes underwent separate statistical analyses. Depending on their distribution, as determined by the Shapiro-Wilk test, we described numerical data as means S.D. or median and interquartile range (IQR). We also reported categorical data using numbers and percentages. The Mann-Whitney U test was used to compare the irregularly distributed numerical data, while the Chi-square test was applied to investigate the association between the categorical variables. Alternatively, Fisher’s Exact or Monte Carlo correction test was applied when more than 20% of the cells had an expected count of less than five. Univariate and multivariate logistic regression analyses evaluated the predictors of different HT subtypes. The significance of the obtained results was judged at the 5% level.

## Results

**Fig. 1:**
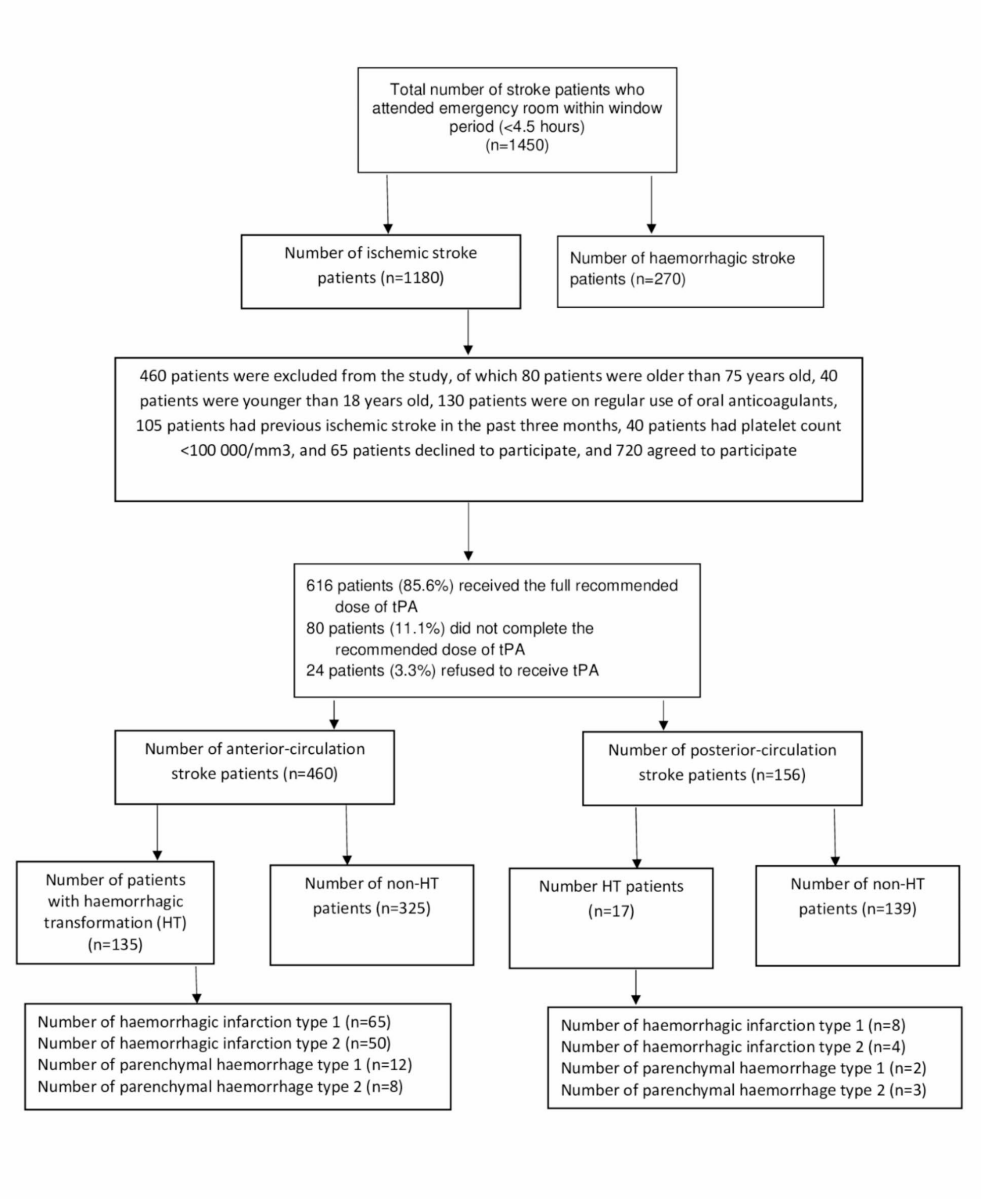
Study flow diagram.

Overall, we screened 1450 patients who came to the emergency with clinical manifestations of stroke. One thousand one hundred eighty patients had an ischemic stroke, of which 460 patients were excluded from the study, as 80 patients were older than 75 years old, 40 patients were younger than 18 years old, 130 patients were on regular use of oral anticoagulants, 105 patients had a previous ischemic stroke in the past three months, 40 patients had platelet count < 100 000/mm3 and 65 patients declined to participate, and 720 agreed to participate, and 616 patients (232 females & 384 males) received the total recommended dose of alteplase. They were included in the per-protocol analysis; only 152 (24.7%) had HT, 73 patients (11.9%) had hemorrhagic infarction type 1, 54 patients (8.8%) had hemorrhagic infarction type 2, fourteen patients (2.3%) had parenchymal hemorrhage type 1, eleven patients (1.8%) had parenchymal hemorrhage type 2, as shown in Fig. 1.

Regarding the symptomatic clinical deterioration following HT (4-points worsening in NIHSS after treatment compared with baseline)^18^, we found that 27 patients in the hemorrhagic infarction type 1 group, 26 patients in hemorrhagic infarction type 2 group, eleven patients in parenchymal hemorrhage type 1 group, and ten patients in parenchymal hemorrhage type 2 group had symptomatic clinical deterioration with P-value 0.002.

Patients with HI type 1 had statistically significantly higher baseline NIHSS and a higher percentage of anterior circulation stroke with P-values < 0.001 and 0.02, respectively. In comparison, Patients with HI type 2 had statistically significantly higher baseline NIHSS with a P-value < 0.001 as shown in Table 1.

**Table 1:** Patients’ baseline characters HI type1 and type2.

Demographic data	(Non-HT group) *N* = 464	Hemorrhagic infarction type 1, *N* = 73	*P*-value	Hemorrhagic infarction type 2, *N* = 54	*P*-value
Gender, no, (percentage)					
Male, no, (percentage)	288.0 (62.1%)	43.0 (58.9%)	0.61	35.0 (64.8%)	0.69
Age at time of presentation, median (IQR)	61.0 (58.0–66.0)	60.0 (55.0–66.0)	0.40	60.0 (57.0–65.0)	0.20
Location of infarction, no, (percentage)					
Anterior circulation, no, (percentage)	320.0 (69%)	60.0 (82.2%)	0.02^*^	43.0 (79.6%)	0.11
NIHSS at time of admission, median (IQR)	10 (7.3–17.0)	17.0 (14.0–20.0)	< 0.001^*^	17.0 (12.0–20.0)	< 0.001^*^
Door to needle time (min.), median (IQR)	60.0 (55.0–66.0)	55.0 (55.0–66.0)	0.45	55.0 (55.0–66.0)	0.46
Time of receiving IV rtPA from stroke onset (min.), median (IQR)	174.0 (163.0–200.0)	174.0 (164–200)	0.48	175.0 (162–200)	0.58
Patients on antiplatelets before stroke					
Aspirin and clopidogrel	120.0 (25.9%)	17.0 (23.3%)	0.64	12.0 (22.2%)	0.56
Aspirin	114.0 (24.6%)	20.0 (27.4%)	0.60	15.0 (27.8%)	0.61
Clopidogrel	90.0 (19.4%)	17.0 (23.3%)	0.44	14.0 (25.9%)	0.15
Ticagrelor	15.0 (3.2%)	2.0 (2.7%)	0.82	2.0 (3.7%)	0.85
Patients on anticoagulants before stroke					
Warfarin	18.0 (3.9%)	3.0 (4.1%)	0.93	2.0 (3.7%)	0.95
Rivaroxaban	14.0 (3.0%)	2.0 (3.0%)	0.90	1.0 (1.9%)	0.63
Apixaban	10.0 (2.2%)	1.0 (1.4%)	0.66	0	

*IQR* inter-quartile range, *p* p value, *Statistically significant at *p* ≤ 0.05, *HI* hemorrhagic infarction.

Regarding the analysis of the different risk factors, patients with HI type1 and 2 had a statistically significant higher percentage of atrial fibrillation, large-vessel stroke, and cardio-embolic stroke with a P-value of < 0.001, < 0.001, < 0.001, < 0.001, < 0.001, and < 0.001, respectively and a statistically significant lower percentage of small-vessel stroke with P-value 0.002& 0.003, respectively as shown in Table 2.

**Table 2:** Comparison between the non-HT patients and HI type 1 and 2 patients’ risk factors.

Risk factor, no, (percentage)	Non-HT group, *N* = 464	HI type 1, *N* = 73	*P*-value	HI type 2, *N* = 54	*P*-value
Hyperlipidemia	156.0 (33.6%)	24.0 (32.9%)	0.90	18.0 (33.3%)	0.13
Diabetes mellitus	164.0 (35.3%)	25.0 (34.2%)	0.86	20.0 (37.0%)	0.81
Hypertension	288.0 (62.1%)	47.0 (64.4%)	0.70	32.0 (59.3%)	0.69
Atrial fibrillation	67.0 (14.4%)	25.0 (34.2%)	< 0.001^*^	20.0 (37.0%)	< 0.001^*^
Ischemic heart disease	57.0 (12.3%)	7.0 (9.6%)	0.51	5.0 (9.3%)	0.52
Admission hyperglycemia	140.0 (30.2%)	25.0 (34.2%)	0.48	19.0 (35.2%)	0.45
Previous TIA	131.0 (28.2%)	19.0 (26.0%)	0.70	15.0 (27.8%)	0.94
Etiology, no, (percentage)					
Large artery atherosclerosis	68.0 (14.7%)	25.0 (34.2%)	< 0.001^*^	20.0 (37.0%)	< 0.001^*^
Cardio-embolism	55.0 (11.9%)	36.0 (49.3%)	< 0.001^*^	28.0 (51.9%)	< 0.001^*^
Small artery occlusion	152.0 (32.8%)	11.0 (15.1%)	0.002^*^	7.0 (13.0%)	0.003^*^
Stroke of undetermined etiology	136.0 (29.3%)	26.0 (35.6%)	0.28	20.0 (37.0%)	0.24
Co-morbid conditions on admission					
Temperature on admission, median (IQR)	37.4 (37.2–37.8)	37.6 (37.3–37.9 )	0.41	37.6 (37.3–38.0)	0.36
Cholesterol level on admission, mg/dL, median (IQR)	188.0 (181.0–237.0)	194.0 (187.0–246.0)	0.12	190.0 (189.0–245.0)	0.31
Mean value of two points evaluation of BP within 24 h of symptoms onset, mmHg, median (IQR)	145.0 (130.0–165.0)	150.0 (135.0–165.0)	0.38	150.0 (135.0–165.0)	0.17

*HI* hemorrhagic infarction, *IQR* inter-quartile range, *p* p value, *Statistically significant at *p* ≤ 0.05.

We evaluated the relative contribution of the different variables to different subtypes of HT. We found that in univariate analysis, some factors had statistically significant predictive values of HI type 1 as follows: female gender (P-value 0.02), older age at presentation (P-value 0.03), baseline NIHSS on admission (P-value < 0.001), anterior-circulation stroke (P-value < 0.001), cardioembolic stroke (*P* < 0.001), atrial fibrillation (P-value < 0.001), a stroke of undetermined aetiology (*P* = 0.018), and large-vessel stroke (*P* = 0.02). However, a multivariate regression model revealed that baseline NIHSS score (odds ratio [OR], 1.20; 95% CI, 1.18 to 1.21; *P* < 0.001), anterior-circulation stroke (OR, 11.04; 95% CI, 9.81 to 12.70; *P* < 0.001), and cardio-embolic stroke (OR, 1.69; 95%, CI, 1.62 to 1.76; *P* < 0.001), independently predict HI type1, as shown in Table 3.

**Table 3:** Univariate and multivariate logistic regression analysis of the characters and risk factors of patients with HI type1 (*n* = 73).

	*P*	Univariate, OR (LL–UL 95% C.I)	*p*	^#^Multivariate, OR (LL–UL 95% C.I)
Females	0.02^*^	0.71* (0.67–0.76)	0.093	
Age at time of presentation	0.03^*^	1.02* (1.019–1.026)	0.072	
Lesion location (anterior circulation)	< 0.001^*^	1.84* (1.70–2.00)	< 0.001^*^	11.04^*^ (9.81–12.70)
NIHSS at time of admission	< 0.001^*^	1.23* (1.22–1.54)	< 0.001^*^	1.196*(1.178–1.209)
Admission hyperglycemia	0.32	1.04 (0.97–1.11)		
Door to needle time (min)	0.388	1.002 (0.997–1.008)		
Time of receiving IV rtPA from stroke onset	0.514	0.998 (0.989–1.007)		
Hyperlipidemia	0.617	1.152 (0.627–2.474)		
Diabetes mellitus	0.534	0.982 (0.469–2.100)		
Hypertension	0.471	1.087 (0.509–2.318)		
Previous TIA	0.513	0.812 (0.669–1.817)		
Atrial fibrillation	< 0.001^*^	8.314* (3.127–21.147)	< 0.001^*^	1.689^*^ (1.617–1.761)
Ischemic heart disease	0.317	0.626 (0.147–1.950)		
Large artery atherosclerosis	0.02^*^	2.028* (1.401–5.039)	0.072	7.657 (0.867–9.341)
Cardio -embolism	< 0.001^*^	8.314* (3.128–20.788)	< 0.001^*^	1.689^*^ (1.617–1.761)
Small artery occlusion	0.421	0.787 (0.412–1.060)		
Stroke of undetermined etiology	0.018^*^	0.922 (0.862–0.986)	0.436	2.531 (0.354–6.247)
Temperature on admission	0.124	0.848 (0.841–1.247)		
Cholesterol level on admission	0.512	1.213 (0.714–2.354)		
Mean value of two points evaluation of BP within 24 h of symptoms onset	0.431	1.026 (0.514–2.417)		

*OR* odd’s ratio, *HI* hemorrhagic infarction, *C.I* confidence interval, *LL* lower limit, *UL* upper limit, *CAD* coronary artery disease.

^#^All variables with *p* < 0.05 was included in the multivariate. *Statistically significant at *p* ≤ 0.05.

We also found that in univariate analysis, some factors had statistically significant predictive value of HI type 2 as follows: older age at presentation (P-value 0.08), baseline NIHSS on admission (P-value < 0.001), anterior-circulation stroke (P-value < 0.001), cardioembolic stroke (*P* < 0.001), atrial fibrillation (P-value < 0.001). However, a multivariate regression model revealed that baseline NIHSS score (odds ratio [OR], 1.30; 95% CI, 1.28 to 1.32; *P* < 0.001), anterior-circulation stroke (OR, 11.89; 95% CI, 9.79 to 14.44; *P* < 0.001) cardio-embolic stroke (OR, 1.59; 95%, CI, 1.42 to 1.86; *P* < 0.001), independently predict HI type2, as shown in Table 4.

**Table 4:** Univariate and multivariate logistic regression analysis of the characters and risk factors of patients with HI type2 (*n* = 54).

	*P*	Univariate, OR (LL–UL 95% C.I)	*p*	^#^Multivariate, OR (LL–UL 95% C.I)
Females	0.222	1.049 (0.972–1.131)		
Age at time of presentation	0.08	0.922 (0.818–1.026)		
Lesion location (anterior circulation)	< 0.001^*^	0.660* (0.604–0.720)	< 0.001^*^	11.889^*^ (9.79–14.44)
NIHSS at time of admission	< 0.001^*^	1.224* (1.213–1.235)	< 0.001^*^	1.302* (1.283–1.322)
Admission hyperglycemia	0.07	1.04 (0.97–1.11)		
Door to needle time (min)	0.066	1.006 (1.000–1.012)		
Time of receiving IV rtPA from stroke onset	0.321	0.814 (0.725–1.272)		
Hyperlipidemia	0.537	1.272 (0.717–2.574)		
Diabetes mellitus	0.334	0.782 (0.569–2.230)		
Hypertension	0.357	1.041 (0.609–2.618)		
Previous TIA	0.413	0.812 (0.669–1.817)		
Atrial fibrillation	< 0.001^*^	8.314* (3.127–21.147)	< 0.001^*^	1.689^*^ (1.617–1.761)
Ischemic heart disease	0.137	0.475 (0.347–1.550)		
Admission hyperglycemia	0.09	1.418 (0.837–2.261)		
Large artery atherosclerosis	0.08	1.528 (0.947–2.471)		
Cardio -embolism	< 0.001^*^	8.114* (3.158–18.988)	< 0.001^*^	1.589^*^ (1.417–1.861)
Small artery occlusion	0.068	0.922 (0.862–1.346)		
Stroke of undetermined etiology	0.068	0.942 (0.64–1.56)		
Temperature on admission	0.113	0.782 (0.912–1.357)		
Cholesterol level on admission	0.411	1.312 (0.824–2.244)		
Mean value of two points evaluation of BP within 24 h of symptoms onset	0.451	1.014 (0.614–2.357)		

*OR* odd’s ratio, *HI* hemorrhagic infarction, *C.I* confidence interval, *LL* lower limit, *UL* upper limit, *CAD* coronary artery disease.

^#^All variables with *p* < 0.05 was included in the multivariate. *Statistically significant at *p* ≤ 0.05.

Patients with PH type 1 and type 2 had statistically significant higher baseline NIHSS with P-values < 0.001& <0.001, respectively, as shown in Table 5.

**Table 5:** Patients’ baseline characters in parenchymal hematoma (PH) type1 and type2.

Demographic data	(Non-HT group), *N* = 464	Parenchymal hematoma 1, *N* = 14	*P*-value	Parenchymal hematoma 2, *N* = 11	*P*-value
Gender, no, (percentage)					
Male, no, (percentage)	288.0 (62.1%)	9.0 (64.3%)	0.87	8.0 (72.7%)	0.47
Age at time of presentation, median (IQR)	61.0 (58.0–66.0)	59.0 (59.0–65.0)	0.52	59.0 (58.0–70.0)	0.40
Location of infarction, no, (percentage)					
Anterior circulation, no, (percentage)	320.0 (69%)	11.0 (78.6%)	0.44	9.0 (81.8%)	0.36
NIHSS at time of admission, median (IQR)	10 (7.3–17.0)	18.0 (15.0–20.0)	< 0.001^*^	17.0 (14.0–20.0)	< 0.001^*^
Door to needle time (min.), median (IQR)	60.0 (55.0–66.0)	55.0 (50.0–67.0)	0.50	59.0 (50.0–69.0)	0.51
Time of receiving IV rtPA from stroke onset (min.), median (IQR)	174.0 (163.0–200.0)	175.0 (155.0-200.0)	0.28	175.0 (155.0-200.0)	0.63
Patients on antiplatelets before stroke					
Aspirin and clopidogrel	120.0 (25.9%)	4.0 (28.6%)	0.82	2.0 (18.2%)	0.56
Aspirin	114.0 (24.6%)	4.0 (28.6%)	0.69	3.0 (27.3%)	0.84
Clopidogrel	90.0 (19.4%)	2.0 (14.3%)	0.63	2.0 (18.2%)	0.52
Ticagrelor	15.0 (3.2%)	1.0 (7.1%)	0.44	1.0 (9.1%)	0.30
Patients on anticoagulants before stroke					
Warfarin	18.0 (3.9%)	1.0 (7.1%)	0.54	1.0 (9.1%)	0.38
Rivaroxaban	14.0 (3.0%)	1.0 (7.1%)	0.38	1.0 (9.1%)	0.26
Apixaban	10.0 (2.2%)	0		1.0 (9.1%)	0.13

*IQR* inter-quartile range, *p* p value, *Statistically significant at *p* ≤ 0.05, *PH* parenchymal hematoma.

Patients with PH type1 and 2 had a statistically significant higher percentage of atrial fibrillation and cardio-embolic stroke with a P-value 0.03& 0.04& < 0.001& <0.001, respectively as shown in Table 6.

**Table 6:** Comparison between the non-HT patients and PH patients’ risk factors.

Risk factor, no, (percentage)	(Non-HT group), *N* = 464	Parenchymal hematoma 1, *N* = 14	*P*-value	Parenchymal hematoma 2, *N* = 11	*P*-value
Hyperlipidemia	156.0 (33.6%)	4.0 (28.6%)	0.70	3.0 (27.3%)	0.66
Diabetes mellitus	164.0 (35.3%)	5.0 (35.7%)	0.98	4.0 (28.6%)	0.94
Hypertension	288.0 (62.1%)	9.0 (64.3%)	0.87	7.0 (63.6%)	0.92
Atrial fibrillation	67.0 (14.4%)	5.0 (35.7%)	0.03^*^	4.0 (28.6%)	0.04^*^
Ischemic heart disease	57.0 (12.3%)	2.0 (14.3%)	0.82	1.0 (9.1%)	0.75
Admission hyperglycemia	140.0 (30.2%)	5.0 (35.7%)	0.66	4.0 (28.6%)	0.66
Previous TIA	131.0 (28.2%)	4.0 (28.6%)	0.98	3.0 (27.3%)	0.94
Etiology, no, (percentage)					
Large artery atherosclerosis	68.0 (14.7%)	4.0 (28.6%)	0.15	3.0 (27.3%)	0.25
Cardio-embolism	55.0 (11.9%)	6.0 (42.9%)	0.001^*^	5.0 (45.5%)	0.001^*^
Small artery occlusion	152.0 (32.8%)	2.0 (14.3%)	0.15	1.0 (9.1%)	0.10
Stroke of undetermined etiology	136.0 (29.3%)	6.0 (42.9%)	0.27	5.0 (45.5%)	0.25
Co-morbid conditions on admission					
Temperature on admission, ºC, median (IQR)	37.4 (37.2–37.8)	37.7 (37.3–38.0)	0.18	37.7 (37.3–38.0)	0.14
Cholesterol level on admission, mg/dL, median (IQR)	188.0 (181.0–237.0)	201.0 (192.0–242.0)	0.21	193.0 (190.0–240.0)	0.24
Mean value of two points evaluation of BP within 24 h of symptoms onset, mmHg, median (IQR)	145.0 (130.0–165.0)	155.0 (135.0–165.0)	0.27	160.0 (140.0–165.0)	0.14

*p* p value, *PH* parenchymal hematoma, *IQR* inter-quartile range. *Statistically significant at *p* ≤ 0.05.

In addition, we found that, in univariate analysis, some factors had statistically significant predictive values of PH type1 as follows: older age at presentation (P-value 0.007), baseline NIHSS on admission (P-value < 0.001), cardioembolic stroke (P-value < 0.001), atrial fibrillation (P-value < 0.001). However, a multivariate regression model revealed that baseline NIHSS score (odds ratio [OR], 1.40; 95% CI, 1.55 to 1.81; *P* < 0.001), older age (OR, 1.31; 95% CI, 1.25 to 1.91; P-value 0.02) cardio-embolic stroke (OR, 1.429; 95% CI, 1.517 to 1.941; *P* < 0.001), independently predict PH type1, as shown in Table 7.

**Table 7:** Univariate and multivariate logistic regression analysis of the characters and risk factors of patients with PH 1 (*n* = 14).

	*P*	Univariate, OR (LL–UL 95% C.I)	*p*	^#^Multivariate, OR (LL–UL 95% C.I)
Females	0.220	1.093 (0.949–1.259)		
Age at time of presentation	0.007^*^	1.414* (1.279–1.729)	0.02*	1.312* (1.245–1.912)
Lesion location (anterior circulation)	0.09	1.062 (0.871–1.314)		
NIHSS at time of admission	< 0.001^*^	1.304* (1.279–1.329)	< 0.001^*^	1.402* (1.545–1.812)
Admission hyperglycemia	0.07	1.04 (0.97–1.11)		
Door to needle time (min)	0.066	1.006 (1.000–1.012)		
Time of receiving IV rtPA from stroke onset	0.321	0.814 (0.725–1.272)		
Hyperlipidemia	0.327	1.482 (0.817–1.974)		
Diabetes mellitus	0.393	1.063 (0.924–1.224)		
Hypertension	0.174	1.021 (0.727–1.938)		
Previous TIA	0.641	0.712 (0.642–1.719)		
Atrial fibrillation	< 0.001^*^	9.114* (4.137–22.167)	< 0.001^*^	1.689^*^ (1.617–1.761)
Ischemic heart disease	0.137	0.475 (0.347–1.550)		
Admission hyperglycemia	0.21	1.128 (0.847–1.971)		
Large artery atherosclerosis	0.13	1.438 (0.947–2.171)		
Cardio -embolism	< 0.001^*^	8.834* (3.128–17.968)	< 0.001^*^	1.429^*^ (1.517–1.941)
Small artery occlusion	0.128	0.712 (0.962–1.646)		
Temperature on admission	0.183	0.817 (0.911–1.129)		
Cholesterol level on admission	0.462	1.343 (0.714–2.184)		
Mean value of two points evaluation of BP within 24 h of symptoms onset	0.542	1.286 (0.753–2.713)		

*OR* odd’s ratio, *PH* parenchymal hematoma, *C.I* confidence interval, *LL* lower limit, *UL* upper limit, *CAD* coronary artery disease.

^#^All variables with *p* < 0.05 was included in the multivariate. *Statistically significant at *p* ≤ 0.05.

We also found that in univariate analysis, some factors had statistically significant predictive values of PH type2 as follows: baseline NIHSS on admission (P-value < 0.001), cardioembolic stroke (*P* < 0.001), and atrial fibrillation (P-value < 0.001). However, a multivariate regression model revealed that baseline NIHSS score (odds ratio [OR], 1.312; 95% CI, 1.645 to 1.932; *P* < 0.001), cardio-embolic stroke (OR, 1.329; 95%, CI, 1.417 to 1.951; *P* < 0.001), independently predict PH type2, as shown in Table 8.

**Table 8:** Univariate and multivariate logistic regression analysis of the characters and risk factors of patients with PH 2 (*n* = 11).

	*P*	Univariate, OR (LL–UL 95% C. I)	*p*	^#^Multivariate, OR (LL–UL 95%C.I)
Females	0.310	1.083 (0.849–1.314)		
Age at time of presentation	0.08	1.172 (1.119–1.529)		
Lesion location (anterior circulation)	0.07	1.112 (0.807–1.427)		
NIHSS at time of admission	< 0.001^*^	1.134* (1.242–1.412)	< 0.001^*^	1.312* (1.645–1.932)
Admission hyperglycemia	0.112	1.09 (0.95–1.21)		
Door to needle time (min)	0.086	1.008 (0.917–1.132)		
Time of receiving IV rtPA from stroke onset	0.211	0.874 (0.716–1.322)		
Hyperlipidemia	0.127	1.322 (0.717–1.734)		
Diabetes mellitus	0.273	1.043 (0.914–1.324)		
Hypertension	0.244	1.051 (0.711–1.728)		
Previous TIA	0.571	0.781 (0.742–1.763)		
Atrial fibrillation	< 0.001^*^	7.164* (3.437–20.785)	< 0.001^*^	1.649^*^ (1.817–1.981)
Ischemic heart disease	0.149	0.412 (0.317–1.452)		
Admission hyperglycemia	0.208	1.168 (0.811–1.751)		
Large artery atherosclerosis	0.152	1.511 (0.913–2.153)		
Cardio -embolism	< 0.001^*^	8.114* (5.128–14.958)	< 0.001^*^	1.329^*^ (1.417–1.951)
Small artery occlusion	0.211	0.752 (0.912–1.516)		
Stroke of undetermined etiology	0.261	1.071 (0.934–1.219)		
Temperature on admission	0.197	1.048 (0.934–1.318)		
Cholesterol level on admission	0.537	1.353 (0.714–2.493)		
Mean value of two points evaluation of BP within 24 h of symptoms onset	0.348	1.173 (0.514–2.278)		

*OR* odd’s ratio, *PH* parenchymal hematoma, *C.I* confidence interval, *LL* lower limit, *UL* upper limit, *CAD* coronary artery disease.

^#^All variables with *p* < 0.05 was included in the multivariate, *Statistically significant at *p* ≤ 0.05.

Regarding the mortality among our patients, we found that 22 (14.5%) patients among those who experienced hemorrhagic transformation died during their hospital stay, of which one patient died due to recurrent ischemic stroke, one due to brain oedema, one due to seizures, seven due to haemorrhagic infarction, five due to pneumonia, two due to pulmonary embolism, three due to myocardial infarction, one due to acute renal failure, and one due to orolingual oedema, also 32 (6.9%) patients in the non-HT group patients died during their hospital stay of which three patients died due to recurrent stroke, three due to brain oedema, three due to seizures, eight due to pneumonia, four due to pulmonary embolism, seven due to myocardial infarction, three due to acute renal failure, and one due to orolingual oedema, (HR 2.38; 95% CI, 1.36–4.17; P-value = 0.003) as shown in Table 9.

**Table 9:** Mortality rates and causes during hospital stay in HT and non-HT groups.

Death causes	(Non-HT group) *N* = 464	(HT group) *N* = 152	Hazard ratio (95% CI)	*P*-value
All causes of death	32.0 (6.9%)	22.0 (14.5%)	2.38 (1.36–4.17)	0.003*
Neurological mortality causes				
Recurrent ischemic stroke	3.0 (0.6%)	1.0 (0.7%)		
Brain edema	3.0 (0.6%)	1.0 (0.7%)		
Hemorrhagic infarction	0.0	7.0 (8.6%)		
Seizures	3.0 (0.6%)	1.0 (0.7%)		
Non-neurological mortality causes				
Pneumonia	8.0 (1.7%)	5.0 (3.3%)		
Pulmonary embolism	4.0 (0.9%)	2.0 (1.3%)		
Myocardial infarction	7.0 (1.5%)	3.0 (2.0%)		
Acute renal failure	3.0 (0.6%)	1.0 (0.7%)		
Orolingual edema	1.0 (0.2%)	1.0 (0.7%)		

*CI* confidence interval, *p* value, *HT* hemorrhagic transformation. *Statistically significant at *p* ≤ 0.05.

## Discussion

Although alteplase is the only FDA-approved treatment for acute stroke, it could lead to HT^19,20^, and the global prevalence of HT in patients treated with alteplase was 32%. In comparison, in East Asia, 35%, and in non-east Asia, 31%^4^; however, most studies evaluated the post-alteplase haemorrhagic transformation of brain infarction as a homogeneous entity and the factors that contributed to haemorrhagic infarction subtypes in the Arab population are poorly understood, so we conducted our study to be the first-ever multicentre prospective study that evaluated the predictors of each subtype of post-alteplase haemorrhagic infarction in the Middle East.

Our study’s primary outcome was the HT1 as it was the commonest hemorrhagic transformation^21,22^. while our secondary outcome included the other types of hemorrhagic transformation.

In our study, 24.7% of the patients had a haemorrhagic transformation, and this agrees with the findings of Strbian et al. and Sun et al.^9,23^, who stated that up to 30% of AIS patients who were treated with tPA had haemorrhagic transformation. Most of the HT was asymptomatic, but in our study, 75 patients (12.2%) had symptomatic worsening due to HT.

Our analysis showed that in the Arab population from Egypt and Saudi Arabia, gender and door-to-needle time (DTN) were not predictive factors of any HT subtypes in AIS patients treated with tPA; our findings agreed with Lansberg et al., Liu et al., Nisar et al., and Sun et al.^9,24–26^, who stated that gender, DTN did not predict haemorrhagic transformation in AIS patients treated with tPA, and disagreed with the findings of Chenna et al. and Xue et al.^27,28^, who found that DTN was an independent predictor of haemorrhagic transformation in AIS patients treated with tPA.

Our finding that DTN time did not predict the haemorrhagic transformation in AIS patients may be attributed to the fact that in our HT group, the median time from onset to thrombolysis was 180 min, which was shorter than the median time from onset to thrombolysis in the study of Chenna et al.^27^ which was 227 min. The median DTN time in our patients was 55 min shorter than the median DTN time in the study of Xue et al.^28^, which was 66 min. It is recognized that the longer the time to thrombolysis, the higher the occurrence of the haemorrhagic transformation.

Our study found that older age was not a predictor of HI type 1, HI type 2, and PH type 2. This disagreed with the results of Liu et al. and Sun et al.^9,25^, who found that age ≥ 68 and age ≥ 70, respectively, were predictors of haemorrhagic transformation in AIS patients treated with tPA. Also, our finding that age did not predict the haemorrhagic transformation in HI type 1, HI type 2, and PH type 2 patients can be attributed to the relatively younger mean age of our patients, which was 62 years, and that our study included patients aged younger than 75 years, unlike the mean ages in the study of Liu et al. and Sun et al.^9,25^ which were 67 years and 69 years and the two studies included patients aged up to 80 years. It is known that age *>* 75 years increases the risk of HT.

We found that admission hyperthermia did not predict the haemorrhagic transformation in AIS patients, which disagreed partially with the findings of Leira et al.^29^, who found that high body temperature within the first 24 h after ischemic stroke is a risk factor for HT in patients untreated with rtPA, this could be explained as Leira study included patients who did not receive alteplase and it is known that patients who received alteplase have different outcomes and complications than those who did not receive alteplase^30^.

In addition, we found that hyperlipidaemia was not a predictor of haemorrhagic transformation in AIS patients, which agreed with the findings of Martínez et al.^31^, who found that total cholesterol level was not associated with increased risk of parenchymal haemorrhagic transformation.

We also found that admission blood pressure could not predict the haemorrhagic transformation in AIS patients, which agreed with van den Berg et al.^32^, who found that elevated systolic blood pressure was not associated with the risk of haemorrhagic transformation.

Also, we found that higher baseline NIHSS was an independent predictor of all Haemorrhagic infarction subtypes. At the same time, anterior-circulation stroke was an independent predictor of HT types 1 and 2, which agreed with Chenna et al., Dornak et al., Sun et al., and Xue et al.^9,27,28,33^.

Regarding the analysis of the relationship between different risk factors and the haemorrhagic transformation, we found that atrial fibrillation (AF) was an independent predictor of all haemorrhagic infarction subtypes; this agreed with the findings of Cronin et al., Tong et al., and Al-Khaled et al.^34–36^ who found that atrial fibrillation, was related to the haemorrhagic transformation in AIS patients.

Concerning the analysis of the relationship between different ischemic stroke aetiologies and the haemorrhagic transformation, we found that cardioembolic stroke was an independent predictor of all haemorrhagic infarction subtypes; this agreed with the findings of Liu et al. and Xue et al.^25,28^.

In our study, we found a significantly higher hospital mortality rate in the HT group due to neurological and non-neurological complications compared to the non-HT group; this agreed with the findings of Kastrup et al.^37^, who found that HT increased post-stroke mortality.

Although our results were encouraging, our study has some limitations. First, the prospective nature of our study led to a relatively small number of patients; second, the study had patients from two Middle Eastern countries; third, we did not include an indicator of white matter lesions burden in our analysis due to the unavailability of a specific MRI software program.

Many aspects need thorough investigation in future studies, such as the assessment of the efficacy and safety of alteplase in patients aged 85 years and older as they have different demographics and risk factors^38^ and identifying the predictors of different types of hemorrhagic transformation in different ethnicities to allow more generalization of the results and evaluating the impact of white matter hyperintensities burden on hemorrhagic transformation post-alteplase, in addition, we need to investigate the predictors of hemorrhagic transformation after alteplase in specific groups of ischemic stroke patients such as atrial fibrillation patients.

## Conclusion

In AIS patients treated with alteplase in Egypt and Saudi Arabia, higher NIHSS, cardioembolic stroke and atrial fibrillation were independent predictors of all ECASS-based subtypes of hemorrhagic infarction; in addition, anterior-circulation stroke was an independent predictor of hemorrhagic infarction type 1 and 2, while older age was also an independent predictor of parenchymal hematoma type1.

## Data Availability

The datasets generated and analyzed during the current study are not publicly available due to the ethical regulations of our university, but are available from the corresponding author (Mohamed G. Zeinhom) on reasonable request.

## Abbreviations

AIS: Acute ischemic stroke
ACS: Anterior-circulation stroke
CNS: Central nervous system
CT: Computerized tomography
ECASS: European cooperative acute stroke study
ECG: Electrocardiogram
FDA: Food and drug administration
HT: Hemorrhagic transformation
INR: International normalization ratio
IQR: Inter quartile range
MRA: Magnetic resonance angiography
MRI: Magnetic resonance imaging
mRS: Modified Rankin scale
NIHSS: National institute of health stroke score
PCS: Posterior-circulation stroke
TPA: Recombinant tissue plasminogen activator
TIA: Transient ischemic attack
